# Intronic Alternative Polyadenylation in the Middle of the *DMD* Gene Produces Half-Size N-Terminal Dystrophin with a Potential Implication of ECG Abnormalities of DMD Patients

**DOI:** 10.3390/ijms21103555

**Published:** 2020-05-18

**Authors:** Abdul Qawee Mahyoob Rani, Tetsushi Yamamoto, Tatsuya Kawaguchi, Kazuhiro Maeta, Hiroyuki Awano, Hisahide Nishio, Masafumi Matsuo

**Affiliations:** 1 Research Center for Locomotion Biology, Kobe Gakuin University, Nishi, Kobe 6512180, Japan; rani@reha.kobegakuin.ac.jp (A.Q.M.R.); maeta@reha.kobegakuin.ac.jp (K.M.); nishio@reha.kobegakuin.ac.jp (H.N.); 2 KNC Department of Nucleic Acid Drug Discovery, Faculty of Rehabilitation, Kobe Gakuin University, Nishi, Kobe 6512180, Japan; 3 Department of Clinical Laboratory, Kobe University Hospital, Chuo, Kobe 6500017, Japan; tetsushi@med.kobe-u.ac.jp; 4 Translational Research Department, Daiichi Sankyo RD Novare Co., Ltd., Edogawa, Tokyo 1348630, Japan; kawaguchi.tatsuya.sg@rdn.daiichisankyo.co.jp; 5 Department of Pediatrics, Kobe University Graduate School of Medicine, Chuo, Kobe 6500017, Japan; awahiro@med.kobe-u.ac.jp; 6 Department of Occupational Therapy, Faculty of Rehabilitation, Kobe Gakuin University, Nishi, Kobe 6512180, Japan

**Keywords:** *DMD*, intronic alternative polyadenylation, dystrophin, Dpm234, Duchenne muscular dystrophy, iPS-derived cardiomyocytes

## Abstract

The *DMD* gene is one of the largest human genes, being composed of 79 exons, and encodes dystrophin Dp427m which is deficient in Duchenne muscular dystrophy (DMD). In some DMD patient, however, small size dystrophin reacting with antibody to N-terminal but not to C-terminal has been identified. The mechanism to produce N-terminal small size dystrophin remains unknown. Intronic polyadenylation is a mechanism that produces a transcript with a new 3′ terminal exon and a C-terminal truncated protein. In this study, intronic alternative polyadenylation was disclosed to occur in the middle of the *DMD* gene and produce the half-size N-terminal dystrophin Dp427m, Dpm234. The 3′-rapid amplification of cDNA ends revealed 421 bp sequence in the downstream of *DMD* exon 41 in U-251 glioblastoma cells. The cloned sequence composing of the 5′ end sequence of intron 41 was decided as the terminal exon, since it encoded poly (A) signal followed by poly (A) stretch. Subsequently, a fragment from *DMD* exon M1 to intron 41 was obtained by PCR amplification. This product was named Dpm234 after its molecular weight. However, Dpm234 was not PCR amplified in human skeletal and cardiac muscles. Remarkably, Dpm234 was PCR amplified in iPS-derived cardiomyocytes. Accordingly, Western blotting of cardiomyocyte proteins showed a band of 234 kDa reacting with dystrophin antibody to N-terminal, but not C-terminal. Clinically, DMD patients with mutations in the Dpm234 coding region were found to have a significantly higher likelihood of two ECG abnormal findings. Intronic alternative splicing was first revealed in Dp427m to produce small size dystrophin.

## 1. Introduction

The *DMD* gene is one the largest genes in humans and spans over 2400 kb on the X-chromosome composing of 79 exons. The full-length transcript encodes dystrophin Dp427m, a slender subsarcolemmal protein consisting of 3685 amino acids separated into four domains, the N-terminal, rod, cysteine-rich and C-terminal domains. Out-of-frame or nonsense mutations in the *DMD* gene cause Duchenne muscular dystrophy (DMD) (OMIM#310200), a fatal progressive muscle wasting disease. DMD is characterized by dystrophin Dp427m deficiency in skeletal muscle. On the other hand, nearly half of DMD patients have been reported to express small size dystrophin reactive with dystrophin antibody to N-terminus, but not to C-terminus [1]. Likewise, a half-size N-terminal dystrophin fragment was identified in a DMD patient who had deletion of exons 42 and 43 [2]. Although these incidents strongly indicated the presence of half-size N-terminal dystrophin, no further study has been conducted on this as far as we know.

The *DMD* gene exhibits a highly complex arrangement and encodes additional alternative promoters/first exons within introns, with transcription from each promoter producing a tissue- or development-specific dystrophin isoform [3,4]. In total, eight alternative promoters drive the expression of four full-length and four short dystrophin isoforms [3,4]. Alternative splicing adds further complexity to the *DMD* transcript [5]. Especially, skipping of penultimate exon 78 is a mechanism to produce Dp427m with different C-terminal amino acid sequence [6]. Recent studies revealed wider varieties of alternative splicing in the *DMD* gene transcript [7,8,9].

Polyadenylation is one of post-transcription modifications to add the poly(A) tail on the 3′ terminus of mRNA, which is fundamental for mRNA stability, nuclear export and efficient translation. The core molecular machinery responsible for the definition of a poly(A) site includes a poly(A) signal present in a pre-mRNA, usually an AAUAAA hexamer. Alternative polyadenylation is a mechanism that generates distinct 3′ termini on mRNA and can be classified into four general classes [10]. Intronic polyadenylation, the lowest class among four, involves the cleaving of pre-mRNA at the cryptic intronic poly(A) signal. Thereby, it produces a transcript with a new 3′ terminal exon and a C-terminal truncated protein. In the *DMD* gene, intronic polyadenylation was shown to occur in intron 70 of the Dp71 transcript [11]. However, intronic polyadenylation has never been identified in Dp427m transcript.

DMD patients show initial muscle weakness at age 3–5 years, with weakness progressing with age and eventually resulting in loss of ambulation by age 12 years. With aging, muscle wasting further progresses to affect respiratory and/or cardiac muscles. Multidisciplinary care has increased the life expectancy of DMD patients from 15–19 to >30 years [12,13]. The increased lifespan of DMD patients has resulted in cardiomyopathy being the key determinant of survival in DMD patients [12,14]. The association of cardiomyopathy with specific mutations in the *DMD* gene remain unclear [15,16]. We recently reported that the expression of Dp116, a dystrophin isoform expressed in a Schwann cell specific manner [17], correlated with early development of cardiac failure in DMD patients [18]. It was indicated that cardiomyopathy is dystrophin isoform specific. Electrocardiographic (ECG) abnormalities are markers of cardiomyopathy in DMD patients [19], being detected in over 90% of these patients [20,21]. Cardiomyopathy in DMD includes a wide variety of rhythm and voltage abnormalities, including sinus tachycardia, short PR intervals, and deep and narrow Q waves [21,22,23,24]. However, the association of ECG abnormalities with isoform deficiencies in DMD patients remains to be determined.

We had analyzed short isoform transcripts in U-251 glioblastoma cells [8,9] and obtained results suggesting expression of a 3′ truncated Dp427m transcript. The present study utilized 3′-rapid amplification of cDNA ends (3′ RACE) to identify a novel 3′ terminal exon, constituting the intron 41 sequence, in U-251 cells. Intronic polyadenylation resulted in the production of a novel transcript, which was detected in induced pluripotent stem cell (iPS)-derived cardiomyocytes, but not in adult hearts. The encoded half-size N-terminal dystrophin isolated from cardiomyocytes was named Dpm234. Although Dpm234 was development specific, Dpm234 deficiency was implicated in ECG abnormalities in DMD patients. Intronic polyadenylation was first identified in Dp427m transcript to produce a novel isoform that correlated with ECG abnormalities in DMD patients.

## 2. Materials and Methods

### 2.1. Cell Studies

#### 2.1.1. Cell Lines

The U-251 glioblastoma cell line was purchased from the Japanese Collection of Research Bioresources (JCRB; Osaka, Japan) and used within one year. Cells were cultured in minimum essential medium (MEM; Gibco Life Technologies, Waltham, MA, USA) supplemented with 10% fetal bovine serum (FBS) (Hyclone, GE Healthcare Life Sciences, Buckinghamshire, UK). iPS-derived MiraCell^®^ cardiomyocytes (Y50015, Takara Bio, Inc., Shiga, Japan) were cultured in MiraCell CM culture medium (Y50013, Takara Bio, Inc.) and iCell^®^ cardiomyocytes^2^ (CMC-100-012-000.5, FUJIFILM Cellular Dynamics, Inc., Osaka, Japan) were cultured in iCell cardiomyocyte maintenance medium (M1003, FUJIFILM Cellular Dynamics, Inc.). All cells were cultured at 37 °C in a 5% CO_2_ humidified incubator.

#### 2.1.2. cDNA Synthesis from Total RNA

Cultured cells were rinsed twice with phosphate buffered saline (PBS, Sigma-Aldrich Co. St. Louis, MO, USA) and collected using Lysis/Binding Buffer of High Pure RNA isolation kits (Roche Diagnostics, Basel, Switzerland). RNA was extracted from cells using High Pure RNA isolation kits (Roche Diagnostics). Total RNA from human adult skeletal and heart muscles was obtained from a human total RNA Master Panel II (Clontech Laboratories, Inc., Mountain View, CA, USA). cDNA was synthesized from 0.5 µg of each total RNA using random primers as described [25].

#### 2.1.3. Cloning of a Novel Transcript by 3′-RACE 

The 3′ end of the Dp427 transcript was cloned by 3′-RACE using a SMARTer™ RACE System (Takara Bio, Inc.) with specific primers on exons 36 and 37 of the *DMD* gene. First-strand cDNA synthesis from U-251 cell RNA was initiated at the poly(A) tail of mRNA using the 3′-RACE CDS primer A (Takara Bio, Inc.), followed by amplification using a gene-specific primer on exon 36 (5′-TTTGACCAGAATGTGGACCA-3′) and a universal amplification primer targeting cDNA complementary to the 3′ end of the mRNA (Takara Bio, Inc.). Semi-nested amplification using an inner gene-specific primer on exon 37 (5′-GCAGCAAACTTGATGGCAAAC-3′) and the universal amplification primer was also performed using the first amplified product as a substrate.

Full length Dpm234 cDNA was PCR amplified using a primer on exon M1 (5′-ATGCTTTGGTGGGAAGAAGTAG-3′) and a primer on intron 41 (5′-TAAGGGGTTTTCCCTGTTATCTG-3′). The integrity and concentration of the cDNA was examined by amplifying glyceraldehyde 3-phosphate dehydrogenase (*GAPDH*) mRNA by RT-PCR as described [26].

PCR reactions were performed in a total volume of 10 µL, containing 1 µL of cDNA, 2 µL of 5× GXL buffer (Takara Bio, Inc.), 0.25 U of PrimeSTAR^®^ GXL DNA polymerase (Takara Bio, Inc.), 500 nM of each primer, and 250 µM dNTPs (Takara Bio, Inc.). The amplification protocol consisted an initial denaturation at 98 °C for 3 min, followed by 30 cycles of denaturation at 98 °C for 10 s, annealing at 60 °C for 0.5 min, and extension at 68 °C for 6 min on a Mastercycler Gradient PCR machine (Eppendorf, Hamburg, Germany). PCR-amplified products were visualized by agarose gel electrophoresis. For sequencing, the amplified products were separately amplified as small fragments [7]. Each amplified product was purified using a QIAGEN gel extraction kit (QIAGEN, Inc., Hilden, Germany) and subjected to Sanger sequencing using a PreMix sequencing system (Greiner Bio-One Co. Ltd., Tokyo, Japan). Clarified sequences were compared with the reference sequences of human dystrophin cDNA (NM_004006.1) and genomic DNA (NG_012232.1).

#### 2.1.4. Protein Sample Preparation

Cultured iPS derived cardiomyocytes were lysed in RIPA buffer (Cell Signaling Technology Inc., Danvers, MA) containing protease inhibitors and sonicated. Fresh frozen human skeletal muscle tissue obtained at autopsy was purchased from Asterand Biosciences (Detroit, MI, USA). Human normal heart lysate (P1234122) was obtained from BioChain (Newark, CA, USA). Tissue samples were disrupted by grinding for 30 s at 2000 rpm twice with a multi-bead Shocker (Yasui Kikai Co. Ltd., Osaka, Japan) in 4% SDS with 4 M urea buffer containing protease inhibitor. After incubation on ice for 20 min, cell lysates and tissue homogenates were centrifuged at 12,000× *g* for 20 min to remove insoluble material. The protein concentrations of the cell lysates and tissue homogenates were determined using Micro BCA Protein Assay kits (Thermo Fisher Scientific, Waltham, MA, USA).

#### 2.1.5. Protein Analysis

Proteins were analyzed by Western blotting. Each protein extract was mixed with one volume of Laemmli sample buffer (Bio-Rad Laboratories, Inc., Hercules, CA, USA) and boiled for 5 min. Precision Plus Protein^TM^ All Blue Standards (Bio-Rad Laboratories) were used as protein size markers. Equal amounts of protein (3 µg) were separated on 4-20% SDS-PAGE gels (Criterion TGX precast Gels, Bio-Rad) and electrotransferred to PVDF membrane using an iBlot2 transfer system (Thermo Fisher Scientific). The membranes were blocked with StartingBlock T20 blocking reagent (Thermo Fisher Scientific) and incubated overnight at 4 ℃ with 1:100 dilutions of antibodies to the N-terminal of dystrophin (NCL-DYSB, Leica Biosystems, Wetzler, Germany), the C-terminal of dystrophin (ab15277, abcam, Cambridge, UK) and a 1:1000 dilution of antibody to vinculin (ab129002, abcam) as a loading control. After washing, the membranes were incubated with a 1:20,000 dilution of secondary HRP-coupled antibody to rabbit for ab15277 and ab129002 (GE Healthcare Life Sciences) and to mouse for NCL-DYSB (GE Healthcare Life Sciences). After washing, the membranes were processed for enhanced chemiluminescence detection using Luminata Forte Western HRP substrate (EMD Millipore, Billerica, MA, USA). Immunoreactive proteins were visualized by Amersham Imager 680RGB (GE Healthcare Life Sciences).

### 2.2. Clinical Studies

#### 2.2.1. Patients

The medical records of 452 male DMD patients at the Department of Pediatrics, Kobe University Hospital, located in the western part of Japan, requiring follow-up between June 1992 and May 2019 were retrospectively reviewed. Of these 452 DMD, 179 fulfilled the following inclusion criteria: (1) who had mutation in the *DMD* gene, (2) whose mutation could be evaluated for isoform deficiency, (3) who underwent routine echocardiography and ECG examinations that were done in the same day between August 2007 and May 2019. The mean age of these 179 DMD patients was 9.6 ± 4.2 years (range: 4–33 years), with each followed-up for a mean 5.2 years from the time of initial echocardiographic evaluation. Each participant underwent blood chemistry tests, and chest radiography at each hospital visit, as described [21]. Echocardiography was scheduled once yearly until age 12 years and biannually thereafter. Patients with abnormal echocardiogram results were started on treatment with an angiotensin-converting enzyme inhibitor, with echocardiography thereafter scheduled biannually even before 12 years of age [27].

#### 2.2.2. ECG Examination

All patients underwent standard 12-lead resting ECG (25 mm/s paper speed, 10 mm/mV amplitude, and 250 Hz sampling rate) using the interpretive ECG-2550 (Nihon Kohden, Tokyo, Japan) with an automated ECAPS12C analyzing system. All ECGs were initially inspected for technical errors and quality, and ECG examination was repeated if necessary. Abnormalities on each ECG were classified according to the Minnesota code classification system, as slightly modified by the Japanese Association for Cerebro-Cardiovascular Disease Control. The rate corrected QT (QTc) interval was calculated as QT/√(60/heart rate).

#### 2.2.3. Echocardiography

All echocardiograms were obtained by a single examiner (T.Y.) with considerable experience in imaging DMD patients. Echocardiography was performed using a commercially available echocardiographic system (Aplio XG; Canon Medical Systems, Tochigi, Japan), with all patients in the supine position. Routine digital grayscale two-dimensional cine loops from three consecutive beats were obtained from the parasternal long-axis, short-axis, and standard apical views. Left ventricular diastolic diameter and left ventricular systolic diameter were obtained from the parasternal long-axis view, as recommended by the American Society of Echocardiography [28]. LVEF was assessed by the modified Simpson method, with cardiac dysfunction defined as an LVEF <53% [28]. Left ventricular dilation was defined as a left ventricular diastolic diameter >55 mm [29].

#### 2.2.4. Statistics

Data were reported as mean ± SD and compared between groups using Welch’s two sample t-test for unpaired data. Proportional differences were evaluated using Fisher’s exact test. Univariate logistic regression analysis was performed to identify factors associated with Dpm234 deficiency, with backward elimination and stepwise variable selection found to yield identical results. Factors with *p*-values ≤0.15 on univariate analysis were entered into a multiple logistic regression model to detect independent determinants of Dpm234 deficiency. Survival curves were estimated by the Kaplan-Meier method and compared by the log-rank test. DMD groups were compared using Cox proportional hazards models. All analyses were performed with commercially available R software version 3.4.1 (R Foundation for Statistical Computing, Vienna, Austria).

#### 2.2.5. Ethics 

The study protocol was approved by the Ethics Committee of the Graduate School of Medicine, Kobe University (approval number 1534). Consent was obtained from patients.

## 3. Results

### 3.1. Cloning of a Novel 3′ end Exon of DMD Transcript by 3′RACE

**Figure 1:**
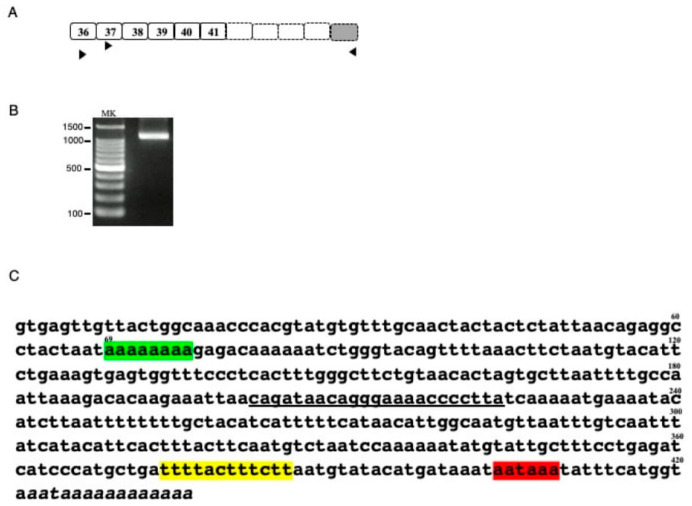
Characterization of the 3′ truncated DMD transcript (**A**) Schematic description of putative 3′ truncated DMD transcript. The exon structure of the putative 3′ truncated DMD transcript is shown schematically. Although previous DMD transcript analysis of U-251 cells suggested a transcript ending at exon 41, exons 42, 43, 44 and 45 were not known. Boxes and numbers in the boxes indicate exons and exon numbers, respectively. Dot lined and shaded boxes indicated unclarified *DMD* and the 3′ terminal exon, respectively. Horizontal arrowheads indicate the location and direction of the universal amplification primer and the exon specific forward primers on exons 36 and 37 used for 3′ RACE. (**B**). Product amplified by 3′ RACE. The semi-nested product amplified by 3′ RACE is shown. A clear band was observed between the 1000 and 1500 bp size markers. MK refers to the size marker (100 bp ladder). (**C**) Sequence of the 3′ RACE product. Sequencing of the 3′ RACE product revealed sequences corresponding to exons 37, 38, 39, 40 and 41. Downstream of exon 41, however, the exon 42 sequence was replaced by an ambiguous nucleotide sequence, as shown. The 421 bp sequence matched the 5′ end sequence of intron 41, with the 3′ end not matching the genomic sequence, indicating a poly(A) tail. In this sequence, however, 2 A nucleotides were missing compared with the reference sequence (green box). The 3′ end contained a polyadenylation signal (aataaa) 13 bp upstream from the poly (A) tail (red box), with a poly U element 19 bp upstream from the poly(A) signal (yellow box). The underlined sequence indicates the binding site for the primer used to amplify the novel transcript.

Characterization of *DMD* transcripts in U-251 glioblastoma cells [8,9] suggested the presence of a *DMD* transcript with its 3′ end in exons 41-45 (data not shown). To explore this finding, the 3′ end of the unknown transcript was cloned by 3′ RACE (Figure 1A). The first PCR amplification using a forward primer on exon 36 and a reverse universal primer did not result in an amplified product. The second PCR using a forward primer on exon 37 and a reverse universal primer revealed a band of a molecular size marker between 1000–1500 bp by agarose gel electrophoresis (Figure 1B). Sequencing of this product revealed intact sequences of exons 37, 38, 39, 40, and 41, but not of exon 42. Rather an undetermined 421 bp sequence and several adenine residues were present at the 3′ end of this transcript (Figure 1C). The 421 bp sequence matched the 5′ end of the intron 41 sequence, with eight adenine residues following nucleotide 69, compared with 10 adenines in the reference sequence (Figure 1C). A consensus polyadenylation signal (aataaa) was identified 13 bp upstream from the poly(A) tail, with a U-rich region located 19 bp upstream of the poly(A) signal. These findings indicated that intronic polyadenylation had created a novel 3′ terminal exon, called exon 41e.

### 3.2. Identification of the Novel DMD Transcript in Cardiomyocytes

**Figure 2:**
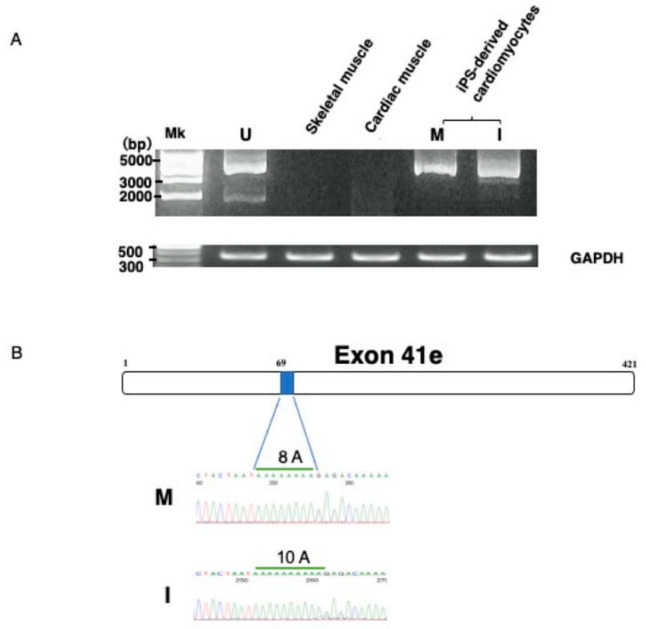
Identification of the novel transcript. (**A**) Amplification of the novel transcript. PCR amplification of the full-length transcript was conducted using a forward primer on exon M1 and a reverse primer on intron 41 (exon 41e). The PCR amplified product of U-251 cDNA was about 5 kbp in size (U). RT-PCR showed that the novel transcript was not expressed by Dp427m expressing skeletal (S) and cardiac (C) muscle tissues. In contrast, amplified products were obtained from iPS-derived MiraCell^®^ cardiomyocytes (M) and iCell^®^ cardiomyocytes^2^ (I). The quality of the prepared cDNA was examined by amplifying *GAPDH* mRNA, yielding a product of expected size obtained from all cDNAs. (**B**) Nucleotide sequences of exon 41e. A part of nucleotide sequences of exon 41e from MiraCell^®^ cardiomyocytes and iCell^®^ cardiomyocytes^2^ is shown (lower). Sequencing of their amplified products showed differences in the numbers of A nucleotides at nucleotide 69, with eight in MiraCell^®^ cardiomyocytes (M) and 10 in iCell^®^ cardiomyocytes^2^ (I). The box shows the schematic structure of exon 41e and the shaded box shows the region of 8A residues in U-251 cells (upper).

To confirm that exon 41e was the 3′ terminal exon of the transcript from the Dp427m promoter, the full-length transcript extending from Dp427m-specific exon M1 to exon 41e was PCR amplified in U-251 cells using primers on the respective exons. A nearly 5 kb sized product was obtained (Figure 2A). Sequencing of this product revealed all 42 exons (data not shown), indicating that a novel shortened Dp427m transcript was expressed in U-251 cells by a mechanism involving intronic polyadenylation. It was afraid that the novel shortened Dp427m transcript is U-251 glioblastoma cell-specific product. The production of the novel transcript from the Dp427m promoter suggested that this transcript would also be produced in normal skeletal and cardiac muscles, in which the Dp427m promoter is highly activated. However, PCR amplification of cDNA from two tissues failed to produce a product corresponding to the novel transcript (Figure 2A), indicating that this transcript was not compulsorily produced by activation of the Dp427m promoter. Unexpectedly, the novel transcript was PCR amplified from cDNA prepared from iPS-derived MiraCell^®^ cardiomyocytes (Figure 2A). The sequence of the amplified product was identical to that in U-251 cells (Figure 2B) and deposited to the GenBank (MT093189). To further confirm its expression in cardiomyocytes, the transcript in iPS-derived iCell^®^ cardiomyocytes^2^ was PCR amplified (Figure 2A). The sequence of the amplified product differed from the nucleotide sequence of exon 41e and deposited to the GenBank (MT093188). Ten adenosine nucleotides were detected at position 69 in iCell^®^ cardiomyocytes, identical to that in the GenBank sequence (NG_012232.1), whereas eight were detected in MiraCell^®^ cardiomyocytes (Figure 2B). Although the novel transcript was expressed in both types of cardiomyocyte, the difference in their exon 41e sequences indicate a polymorphism.

### 3.3. Protein Products of the Cloned Transcripts

**Figure 3:**
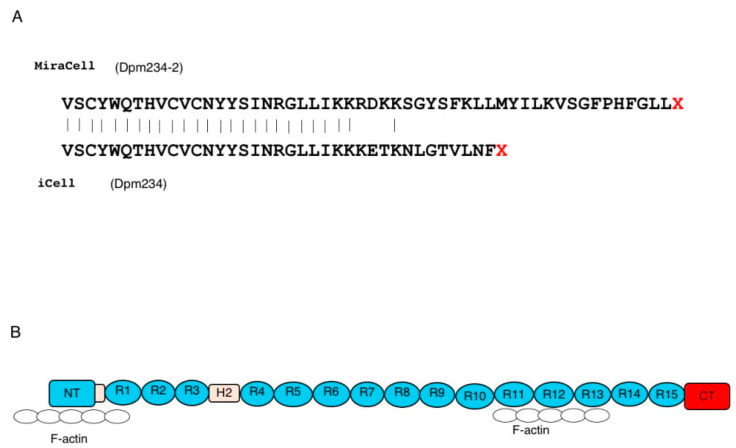
Putative protein product encoded by the novel transcript. (**A**) Presumed amino acid sequences of the two exon 41e sequences. MiraCell^®^ cardiomyocytes contained a 156 bp open reading frame at the 3′ end of the dystrophin reading frame, encoding 52 amino acids (upper), whereas iCell^®^ cardiomyocytes 2 contained a 111 bp open reading frame encoding 37 amino acids (lower). The transcripts of the MiraCell^®^ and iCell^®^ cardiomycocytes encoded putative 235.1 and 233.5 kDa proteins, respectively. Because the latter is consensus sequence, the protein was named Dpm234. To distinguish between them, the 233.5 and 235.1 kDa proteins were named Dpm234 and Dpm234-2, respectively. (**B**) Structure of Dpm234. Schematic depiction of the structure of Dpm234, based on the model of cardiac dystrophin. Dpm234 consisted of an N-terminal domain (blue box), spectrin repeats 1–15 (blue circles), hinges 1 and 2 (yellow boxes) and a specific C-terminal domain (red box). NT and CT in boxes represent N-terminal and C-terminal, respectively. Numbers in boxes and circles represent hinge and repeat numbers, respectively. Open circles represent F-actin that will bind to Dpm234.

The proteins encoded by the cloned transcripts were determined from their nucleotide sequences (Figure 3). Exon 41e from MiraCell^®^ cardiomyocytes contained a 156 bp long open reading frame, encoding 52 additional amino acids, at the 3′ end of the dystrophin reading frame of exon 41. The coding region of this transcript consisted of 6078 bp, encoding 2026 amino acids weighing 235.1 kDa. Exon 41e from iCell^®^ cardiomyocytes^2^, however, contained a 111 bp long open reading frame, encoding 37 additional amino acids, at the 3′ end of exon 41. The coding region of this transcript therefore consisted of 6033 bp, encoding 2011 amino acids, weighing 233.5 kDa. Because the latter sequence had been deposited in GenBank, the size of the protein product was set at 233.5 kDa and the protein was named Dpm234. To distinguish between the two proteins, the 233.5 kDa protein was called Dpm234 and the 235.1 kDa protein was called Dpm234-2. Dpm234 was 15 amino acids shorter than Dpm234-2, with the two sequences differing in eight amino acid residues (Figure 3). The encoded protein therefore included the N-terminal domain, the first and second hinge domains and spectrin repeats 1–15 of Dp427m, but lacked domains necessary to form transmembrane dystrophin-dystroglycan complexes (Figure 3). Thus, Dpm234 was expected to localize to cell membranes through spectrin repeats 1–3 [30] and to bind actin via actin binding motifs located in the N-terminal domain and spectrin repeats 10–13.

### 3.4. Identification of the Dpm234 Protein

**Figure 4:**
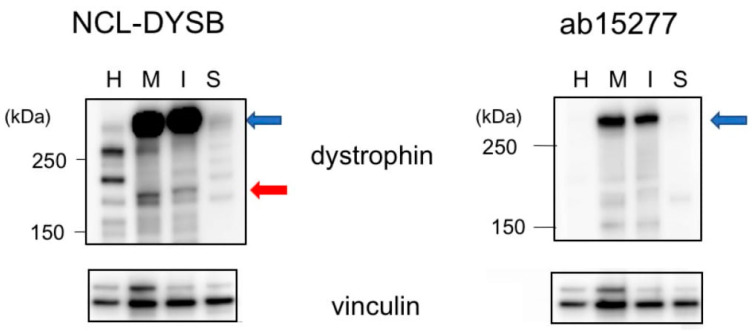
Identification of Dpm234 protein by Western blotting assay. Lysates of iPS-derived MiraCell^®^ cardiomyocytes and iCell^®^ cardiomyocytes^2^, as well as lysates of normal cardiac and skeletal muscles, were analyzed by Western blotting. Antibody to the N-terminal region (NCL-DYSB) of dystrophin yielded a band between 250 kDa and 150 kDa size markers (red arrow) in the lysates of MiraCell^®^ (M) and iCell^®^ (I) cells (left). In contrast, antibody to the C-terminal region of dystrophin (ab15277) did not bind to this band in MiraCell^®^ cardiomyocytes and iCell^®^ cardiomyocytes^2^ (right). Both antibodies, however, bound to Dp427m in cardiac (H) and skeletal (S) muscles (blue arrows). Antibody to vinculin was the loading control (bottom). Size markers are shown on the left side.

It was unclear whether these two newly cloned transcripts were translated into proteins, because their amino acid sequences differed, and they lacked domains necessary to form transmembrane dystrophin-dystroglycan complexes. To determine whether these proteins are expressed, lysates of MiraCell^®^ cardiomyocytes and iCell^®^ cardiomyocytes^2^ were analyzed by Western blotting using an antibody against the dystrophin N-terminal region. Bands between 250 kDa and 150 kDa size markers were present in both cell lysates (Figure 4). These bands were not present when the lysates were blotted with an antibody against the dystrophin C-terminal region. These findings indicated that both Dpm234 and Dpm234-2 were translated into proteins. Western blotting of cardiac muscle and skeletal muscle lysates showed the presence of Dp427m but not Dpm234 (Figure 4), in agreement with transcript analysis in these tissues.

### 3.5. Cardiac Findings in DMD Patients

To assess the clinical significance of Dpm234, ECG and echocardiographic findings were retrospectively analyzed in 179 DMD patients who had been followed up at Kobe University Hospital. Patients were divided into two groups. Of these patients, 67 were Dpm234 deficient, with mutations in exons M1 to 41, and 112 were Dpm234 non-deficient, with mutations in exons 42 to 79. There were no significant between-group differences in age, height, body weight, blood pressure, heart rate, and serum concentrations of creatine kinase and brain natriuretic peptide at both the first and last ECG and echocardiography examinations (Supplementary Table S1). During the observation period, 1035 echocardiograms were obtained from these 179 patients. However, no significant difference was revealed between two groups in echocardiography examinations (Supplementary Table S2). In 1035 ECGs obtained from the 179 DMD patients, 47 types of ECG abnormalities defined by the Minnesota code (MC) were identified (Supplementary Table S3). Univariate analysis of the association of identified codes with Dpm234 deficiency showed that 45 of the 47 abnormalities were not associated with Dpm234 deficiency (data not shown). Interestingly, Dpm234 deficiency was significantly correlated with MC 1-3-3 (0.03 s ≦ Q duration < 0.04 s and R amplitude ≧ 3 mm in lead aVL), observed in seven patients, and MC 8-9 (other arrythmias), observed in 16 patients, with odds ratios (ORs) of 10.9 and 3.1, respectively. Multivariate analysis also found that Dpm234 deficiency correlated significantly with MC 1-3-3 (OR = 10.8; 95% confidence interval [CI] 1.26–93.2; *p* =0.030) and MC 8-9 (OR = 3.08; 95% CI 1.05–9.06; *p* = 0.041). The association of Dpm234 deficiency with rare ECG abnormalities was not unexpected, because Dpm234 expression was development specific. To our knowledge, this study is the first to show that intronic alternative polyadenylation produced half-size N-terminal dystrophin and that specific ECG abnormalities of DMD patients were associated with the deficiency of the small size dystrophin.

## 4. Discussion

This study showed that the *DMD* gene expressed a novel transcript resulting from post-transcriptional intronic polyadenylation. The novel transcript consisted of conventional exons from M1 to 41 and novel exon 41e and encoded an N-terminal half dystrophin Dp427m, Dpm234, lacking the C-terminal half of full-length dystrophin Dp427m. Expression of this transcript was development specific in cardiomyocytes. Because of its limited expression and unique structure, its physiological roles were unclear. However, Dpm234 deficiency was associated with two ECG abnormalities in a large cohort of DMD patients.

Intronic polyadenylation is a mechanism that produces a transcript with a new 3′ terminal exon and a C-terminal truncated protein [31]. Accordingly, Dpm234 acquired novel terminal exon 41e, consisting of sequences in intron 41. Splicing of *DMD* transcript has been shown to proceed step-wise, forming an intermediate exon block [32]. As exon 41 remained unspliced during the first step of splicing [32], it was supposed that intronic polyadenylation occurred in this unspliced intron 41. This was the first intronic polyadenylation observed in the Dp427m transcript, but the second in the *DMD* gene after Dp40 [33]. The specific expression pattern of Dpm234 was compatible with that of other dystrophin isoforms showing tissue or development specificity [4]. Developmental changes in intronic polyadenylation may result from a mechanism of gene regulation [34]. According to this mechanism, intronic polyadenylation is generally activated in immature cells coupled with the upregulation of short genes but is generally suppressed in differentiated cells coupled with the upregulation of long genes. This hypothesis suggests that an intronic polyadenylation product of Dpm234 is present in immature cardiomyocytes [35], but not in differentiated cardiac muscle.

DMD is characterized by many non-muscle complications. Phenotype-genotype analysis of DMD patients has revealed isoform-specific phenotypes. For example, Dp260 deficiencies have been associated with electroretinogram abnormalities, Dp71 deficiencies with cognitive impairment, retinal dysfunction and short stature [36,37,38,39,40], and Dp116 with cardiac dysfunction [18]. The present study showed that Dpm234 was associated with ECG abnormalities. In agreement with this statistical result, biologically supportive results were recently reported, in that low spontaneous firing rate, arrhythmias, and prolonged action potential duration were observed in iPS-derived cardiomyocytes with a nonsense mutation in exon 41 of the *DMD* gene compared with normal cardiomyocytes [41]. These findings clearly indicated that Dpm234 deficient cardiomyocytes have electrophysiological abnormalities. In addition, electrophysiological characteristics observed in iPS-derived cardiomyocytes were observed in heart tissue [42], in agreement with our results showing that cardiomyocyte abnormalities influence cardiac phenotypes. It should be noted, however, that the two ECG abnormalities associated with Dpm234 deficiency are very rare. Future studies are needed to assess ECG abnormalities associated with dystrophin isoforms in DMD patients. Taken together, these results suggested that non-muscle complications of DMD could be explained by specific isoforms. Our result showed that identifying isoforms may facilitate the understanding of complications of DMD.

The function of Dpm234 remains unclear. Intronic polyadenylation has been reported to change protein products from non-secretory to short secretory forms [43]. The intronic polyadenylation production of Dpm234 likely also has a different function from Dp427m, which forms transmembrane complexes. Dpm234 lacks the ability to form transmembrane dystrophin-dystroglycan complexes but possessed sarcoplasma and actin binding domains [30]. Structurally, Dpm234 may play a role in anchoring cortical actin cytoskeletons to membranes.

The inability to identify this novel Dpm234 transcript previously may have been due to the huge size of the *DMD* gene, which hampered extensive analysis of its transcripts, or to the lack of expression of Dpm234 in skeletal muscle, a tissue in which *DMD* transcripts have been analyzed extensively. It should be stressed, however, that half-size N-terminal dystrophin was identified in skeletal muscles of DMD patients by early stage dystrophin studies [1,2]. Particularly, these studies disclosed dystrophins with a size of around 230 kDa that were compatible with the size of Dpm234. Unfortunately, no further study has been conducted at mRNA level for the last three decades. Here, the 3′ RACE successfully resulted in the cloning of the novel 3′ terminal exon and a novel transcript of Dpm234 was cloned in glioblastoma cells. It was disappointing to see non-expression of Dpm234 in normal adult muscle. Considering that half-size dystrophin was identified in DMD patients, it was expected that we could identify both Dpm234 transcript and protein in DMD patients. Further study is needed to confirm this.

This study had several limitations. This study did not analyze the functions of Dpm234, the mechanisms underlying the activation of intronic polyadenylation, or the molecular mechanisms linking Dpm234 deficiency in cardiomyocytes with ECG abnormalities in cardiac muscle. Muscle tissues from DMD patients were unavailable to examine Dpm234 expression.

## Supplementary Materials

The following are available online at https://www.mdpi.com/1422-0067/21/10/3555/s1, Table S1: Demographic characteristics of DMD patients, Table S2: Echocardiographic findings in DMD patients, Table S3: ECG abnormalities identified in DMD patients.
