# Supplementary material for: Intronic Alternative Polyadenylation in the Middle of the DMD Gene Produces Half-Size N-Terminal Dystrophin with a Potential Implication of ECG Abnormalities of DMD Patients
Source: Int J Mol Sci. 2020 May 18;21(10):3555. doi: 10.3390/ijms21103555 (PMC7278912; doi:10.3390/ijms21103555)
Supplement: Supplementary file 1 [file ijms-21-03555-s001.pdf]

International Journal of
Molecular Sciences

MDPI

Supplementary Materials

Intronic Alternative Polyadenylation in the Middle of the DMD Gene Produces Half-Size N-Terminal Dystrophin with a Potential Implication of ECG Abnormalities of DMD Patients

Supplementary Table S1. Demographic characteristics of DMD patients at the time of first (**A**) and last (**B**) examination.

A.

	**Dpm234**		**P value**
	**deficient**	**non-deficient**	
Number of patients	67	112	
Age (year)	9.6±3.6	9.6±4.5	0.42
Height (cm)	128.5±23.4	128.4±18.7	0.53
Weight (kg)	29.0±13.2	28.7±12.7	0.70
Systolic BP (mmHg)	103.6±13.7	100.0±13.3	0.063
Diastolic BP (mmHg)	62.6±10.1	60.6±11.5	0.36
Heart rate (bpm)	91.4±11.8	93.3±12.5	0.20
Creatine kinase (IU/l)	8965±6369	10015±8126	0.83
BNP (pg/ml)	11.7±11.4	11.4±16.2	0.43

B.

	**Dpm234**		**P value**
	**deficient**	**non-deficient**	
Number of patients	67	112	
Age (year)	14.7±5.4	14.8±6.1	0.87
Height (cm)	147.7±16.4	146.4±16.8	0.70
Weight (kg)	42.2±17.1	38.5±15.0	0.16
Systolic BP (mmHg)	103.0±16.2	101.1±12.3	0.33
Diastolic BP (mmHg)	62.6±12.7	61.9±11.8	0.73
Heart rate (bpm)	86.9±15.2	90.3±13.7	0.10
Creatine kinase (IU/l)	3888±4351	4617±5954	0.95
BNP (pg/ml)	26.5±77.3	23.4±45.7	0.99

BP: blood pressure, BNP, brain natriuretic peptide

Supplementary Table S2. Echocardiographic findings in DMD patients at the time of first (A) and last (B) examination.

A

	Dpm234		
	**deficient**	**non-deficient**	**p value**
Ejection fraction (%)	62.9±8.4	63.0±8.9	0.99
Left ventricular end-diastolic dimension (mm)	38.7±4.6	38.2±5.4	0.23
Left ventricular end-systolic dimension (mm)	25.9±4.8	25.3±6.1	0.10
% Fractional shortening (%)	33.3±7.0	34.6±6.4	0.14
Left atrial dimension (mm)	23.8±4.4	23.9±4.4	0.86
Intraventricular septal thickness (mm)	6.4±1.3	6.5±1.3	0.51
Left ventricular posterior wall thickness (mm)	6.5±1.3	6.5±1.1	0.85
Aortic root dimension (mm)	20.8±3.0	21.2±3.5	0.67
Early diastolic wave velocity (cm/s)	97.4±14.7	99.0±17.3	0.23
Atrial wave velocity (cm/s)	47.1±13.2	47.8±13.3	0.82
Early diastolic and atrial wave velocities ratio	2.3±0.8	2.2±0.6	0.79
Early diastolic-wave deceleration time (ms)	157.5±33.3	160.1±29.4	0.43
Inferior vena cava at expiration (mm)	9.7±2.4	9.4±2.3	0.33

B

.	Dpm234		
	**deficient**	**non-deficient**	**p value**
Ejection fraction (%)	52.1±12.0	52.4±12.4	0.83
Left ventricular end-diastolic dimension (mm)	43.2±9.0	42.0±9.2	0.19
Left ventricular end-systolic dimension (mm)	32.3±10.7	31.2±11.3	0.20
% Fractional shortening (%)	26.5±9.0	27.4±10.3	0.45
Left atrial dimension (mm)	23.6±6.2	23.1±6.3	0.60
Intraventricular septal thickness (mm)	7.7±1.5	7.5±1.3	0.90
Left ventricular posterior wall thickness (mm)	7.4±1.4	7.4±1.4	0.95
Aortic root dimension (mm)	20.5±3.1	21.1±3.2	0.12
Early diastolic wave velocity (cm/s)	84.8±17.2	84.2±18.0	0.81
Atrial wave velocity (cm/s)	41.8±10.3	42.6±11.4	0.65
Early diastolic and atrial wave velocities ratio	2.1±0.5	2.1±0.6	0.40
Early diastolic-wave deceleration time (ms)	148.5±28.3	147.0±30.6	0.64
Inferior vena cava at expiration (mm)	11.2±3.4	10.9±3.3	0.66

Supplementary Table S3. ECG abnormalities identified in DMD patients.

	Minnesota code	Patient
Q and QS patterns	1-1-1	38
	1-1-2	6
	1-1-3	3
	1-2-1	66
	1-2-2	15
	1-2-4	1
	1-2-6	17
	1-2-8	1
	1-3-1	73
	1-3-3	7
	1-3-4	1
QRS axis deviation	2-1	4
	2-2	11
	2-3	49
	2-4	4
	2-5	8
High amplitude R waves	3-1	109
	3-2	50
	3-3-1	6
	3-3-2	31
ST junction (J) and segment depression	4-1-2	3
	4-2	9
	4-3	21
	4-4	3
T wave items	5-1	37
	5-2	93
	5-3	34
	5-4	16
	5-5	53
A-V conduction defect	6-4-1	1
	6-5	77
Ventricular conduction defect	7-1	1
	7-2	1
	7-3	16
	7-4	9
	7-5	84
Arrhythmias	8-1-1	8
	8-1-2	2
	8-6	6
	8-7	70
	8-8	1
	8-9	16
Miscellaneous items including ST segment elevation	9-2	100
	9-3-1	6
	9-4-1	166
	9-4-2	26
	9-5	40
